# Health care providers underestimate symptom intensities of cancer patients: A multicenter European study

**DOI:** 10.1186/1477-7525-8-104

**Published:** 2010-09-21

**Authors:** Eivor A Laugsand, Mirjam AG Sprangers, Kristin Bjordal, Frank Skorpen, Stein Kaasa, Pål Klepstad

**Affiliations:** 1 Department of Cancer Research and Molecular Medicine, Faculty of Medicine, Norwegian University of Science and Technology (NTNU), Trondheim, Norway; 2 Department of Medical Psychology, Academic Medical Centre, Amsterdam, The Netherlands; 3 Palliative Care Research Unit and Department of Oncology, Norwegian Radium Hospital, Oslo University Hospital, Montebello, Oslo, Norway; 4 Department of Laboratory Medicine Children's and Women's Health, Faculty of Medicine, Norwegian University of Science and Technology (NTNU), Trondheim, Norway; 5 Department of Oncology, St. Olav's University Hospital, Trondheim, Norway; 6 Department of Circulation and Medical Imaging, Faculty of Medicine, Norwegian University of Science and Technology (NTNU), Trondheim, Norway; 7 Department of Anesthesiology and Emergency Medicine, St. Olav's University Hospital, Trondheim, Norway

## Abstract

**Background:**

Many patients with advanced cancer depend upon health care providers for symptom assessment. The extent of agreement between patient and provider symptom assessments and the association of agreement with demographic- and disease-related factors was examined.

**Methods:**

This cross-sectional study included 1933 patient-health care provider dyads, from 11 European countries. Patients reported symptoms by using the four-point scales of the European Organization of Research and Treatment of Cancer Core Quality of Life Questionnaire (EORTC QLQ-C30) version 3, and providers used corresponding four-point categorical scales. Level of agreement was addressed at the group level (Wilcoxon Signed-Rank test), by difference scores (provider score minus patient score), at the individual level (Intraclass Correlation Coefficients, ICCs) and visually by Bland-Altman plots. Absolute numbers and chi-square tests were used to investigate the relationship between agreement and demographic-, as well as disease-related factors.

**Results:**

The prevalence of symptoms assessed as moderate or severe by patients and providers, respectively, were for pain (67 vs.47%), fatigue (71 vs. 54%), generalized weakness (65 vs. 47%), anorexia (47 vs. 25%), depression (31 vs. 17%), constipation (45 vs. 30%), poor sleep (32 vs. 21%), dyspnea (30 vs. 16%), nausea (27 vs. 14%), vomiting (14 vs. 6%) and diarrhea (14 vs. 6%). Symptom scores were identical or differed by only one response category in the majority of patient-provider assessment pairs (79-93%). Providers underestimated the symptom in approximately one of ten patients and overestimated in 1% of patients. Agreement at the individual level was moderate (ICC 0.38 to 0.59). Patients with low Karnofsky Performance Status, high Mini Mental State-score, hospitalized, recently diagnosed or undergoing opioid titration were at increased risk of symptom underestimation by providers (all p < 0.001). Also, the agreement was significantly associated with drug abuse (p = 0.024), provider profession (p < 0.001), cancer diagnosis (p < 0.001) and country (p < 0.001).

**Conclusions:**

Considerable numbers of health care providers underestimated symptom intensities. Clinicians in cancer care should be aware of the factors characterizing patients at risk of symptom underestimation.

## Background

With the growing incidence of cancer in an aging population, an increasing number of advanced cancer patients are not able to report their symptoms and many depend upon health care providers for symptom assessment [1,2]. Accurate evaluation of symptom intensities is critical for optimal care and ultimately for alleviating symptom burden and improving the quality of life of cancer patients. An inappropriate interpretation of symptoms may lead to overdose of medication (e.g. of an opioid), or conversely may leave the patients undertreated [3,4]. There are several tools for assessment of symptoms in oncology [5,6]. Nevertheless, clinicians have called attention to the lack of consensus about a brief, reliable, bedside symptom assessment tool [7]. The absence of such a tool may be an obstacle for routine assessment, optimal symptom control and ultimately for improvement of quality of life in patients with cancer [3,5,8-10].

Patient ratings of symptoms are generally considered the "gold standard" [11,12]. However, sometimes patients are unable to report on their symptoms, e.g. when they suffer from confusion [13] or communication deficits [11], when the symptom distress is severe [11], or if physical or cognitive disability make them unable to complete assessments [4]. These patients are often excluded from studies and there is no consensus about how their symptoms should be assessed in order to guide clinical decisions. For patients unable to report their symptoms we need to rely on proxy assessments. The usefulness of such proxy reports is dependent on their agreement with patients' report of symptom intensity.

Studies on agreement between patient reports and proxy observations have been performed in a wide range of patient populations [14-16], and in different settings, including palliative care [3,4,17-29]. Studies conducted among cancer patients and their health care providers found a poor and variable agreement between patients and staff [3,17-19,22,24,25]. Some reports have shown that providers tend to underestimate the intensity of physical symptoms, such as pain, and overestimate anxiety, depression and distress [14].

The findings of studies investigating agreement on single symptoms and exploring which clinical factors that cause lack of agreement between self-reports and observer ratings are inconsistent [18,28-30]. Patients' age, gender, tumor site, place of evaluation, Karnofsky performance status, time from admittance into palliative care and time from diagnosis are among the factors studied previously [18,28-30]. Most studies included a limited number of patients, focused on only a few symptoms related to quality of life and were performed in a single department. To use provider reports of patients' symptoms to guide clinical decisions, we need to know which symptoms that can be reliably reported by health care providers and whether certain subgroups of patients are at risk of symptom over- or underestimation by providers.

Therefore, a large-scale study including patients treated with opioids for cancer pain from 11 different European countries was conducted. One of the objectives of the study was to examine the extent of agreement between patient and health care provider assessments, including the association with demographic- and disease-related factors.

## Methods

### Patients

Patients treated in 17 different palliative care centers, outpatient clinics, general or cancer wards in 11 countries were eligible for The European Pharmacogenetic Opioid Study (EPOS), a multicenter, multinational study performed between February 2004 and April 2008. EPOS was designed to study symptoms experienced by cancer pain patients and the pharmacogenetics of opioids in these patients. The examination of agreement on symptom assessments between patients and health care providers was one of the pre-specified objectives of the study. All patients considered for inclusion were 18 years of age or older, had a verified diagnosis of malignant disease and had used a regularly scheduled opioid treatment corresponding to step III at the World Health Organization's analgesic ladder for cancer pain for at least three days. Patients could only participate in the study once. The exclusion criterion was insufficient command of the language spoken in the study center. Patients were informed of the study by their health care provider and gave written informed consent. The study was performed according to the rules of the Helsinki-declaration and approved by each country's or study center's ethical committee.

### Symptom assessments

Patients and health care providers (nurses or physicians) assessed symptoms independently on the same day. The symptoms assessed were pain, fatigue, generalized weakness, anorexia, depression, constipation, sleep disturbance, dyspnea, nausea, vomiting and diarrhea. The health care providers registered symptom severities during the past 24 hours on a four-point verbal rating scale with the descriptors none, mild, moderate and severe. Patients reported their symptoms during the past week by answering the European Organization of Research and Treatment of Cancer Core Quality of Life Questionnaire (EORTC QLQ-C30) version 3 [31]. Symptoms were assessed on a four-point verbal rating scale by the descriptors "not at all", "a little", "quite a bit" and "very much". All symptoms, except fatigue were assessed by single items of the EORTC QLQ-C30. Fatigue was assessed with a scale ranging from zero to 100 including three items: Did you need to rest?, Have you felt weak? and Were you tired?. None of these items were identical with the fatigue item providers responded to. Therefore, the entire fatigue scale was recoded into a four-point scale with increasing symptom intensity from 1 to 4: 0-24 was recoded into one, 25-49 was recoded into two, 50-74 was recoded into three and 75-100 was recoded into four.

### Factors associated with agreement

The possible association between patient-provider agreement and several demographic- and disease-related factors such as age, gender, Karnofsky Performance Status, tumor site, time since diagnosis and affiliation to department is incompletely understood [18,28-30]. The existing literature suggest that agreement might be influenced by such factors (i.e. less agreement as performance status worsens [28], in male patients [29], in younger patients [30], in hospitalized patients [30], in patients with certain cancer diagnoses [30]) but the findings are inconsistent. Therefore, it was decided to perform exploratory analyses of demographic-and disease-related factors thought to be associated with patient-provider agreement based on existing literature and clinical experience. The patient characteristics age, gender, body mass index (BMI), previous or ongoing abuse of alcohol or drugs (yes or no), cancer diagnosis and presence of metastasis were registered by a health care provider. Use of medical treatment including opioids and chemotherapy was obtained from the patients' medical records. The health care providers also noted the time since diagnosis in months, time since start of opioid treatment in months, status of present opioid treatment (as recently initiated and still under titration or as stable dosing) and whether the patient was treated as an inpatient or outpatient. Health care providers assessed cognitive function by performing the Mini Mental State (MMS) examination, scoring the patient between zero and 30, where scores of 23 or less indicated cognitive failure [13,32,33]. In addition, health care providers assessed functional status by the Karnofsky Performance Status, scoring the patient between zero and 100 [34].

### Statistics

Collection and organization of the data were performed by the Pain and Palliation Research Group, Norwegian University of Science and Technology. The statistical software SPSS for Windows version 16.0 was used for all statistical analyses. If data were missing from patients or health care providers on a single symptom, these data were eliminated from analyses involving that variable. No imputations were performed.

Some patients did not report their own symptoms. The definition of not self-reporting was set as answering less than half (< 15) of the questions of the EORTC QLQ-C30. Demographics were reported as absolute numbers, percent, mean and standard deviation (SD). Mann-Whitney U and Fisher's exact tests (for 2 × 2-tables) were used to compare respondents and non-respondents.

The level of agreement between patient- and health care provider assessments was examined by four different approaches. First, agreement at the group level was addressed by the Wilcoxon Signed-Rank test comparing intensity of symptoms as assessed by patients and health care providers. Second, difference scores for each symptom (difference score = health care provider score minus patient score) were calculated to examine good agreement (difference score within ± 1), underestimation (difference score ≤ -2) and overestimation (difference score ≥ 2) by health care providers, compared to patient scores which were considered as the gold standard. Variants of this approach have been used in several previous studies [16,19,27-29].

Third, as a measure of agreement on symptom scores between patients and health care providers at the individual level, intraclass correlation coefficients (ICCs) were computed by a two-way mixed effect model and an absolute agreement definition. ICCs are reported with 95 percent confidence intervals and serve as indicators of chance-corrected agreement at the individual level [35]. Guidelines used for interpretation of ICCs were based on studies demonstrating that for ordinal data ICCs are mathematically equivalent to the weighted kappa statistic [36,37] and the ranges used in previous studies of cancer patient and proxy agreement [17,27,38]: ≤ 0.40 poor to fair agreement, 0.41-0.60 moderate agreement, 0.61-0.80 good agreement and 0.81-1.00 excellent agreement. Fourth, data was examined visually by plotting the differences between the two measures (difference score = health care provider score minus patient score) against their individual means in Bland-Altman plots. These plots are useful for evaluating whether there is any systematic difference between the methods or whether the degree of random variation changes with the mean value [39].

To address whether demographic- and disease-related factors were associated with the agreement between patients and providers, analyses on how these influenced the number and percentage of comparisons where there was good agreement (difference score within ± 1), where the health care provider underestimated (difference score ≤ -2) and overestimated (difference score ≥ 2) were performed [19,27]. Chi-Square tests were used to investigate whether agreement was significantly associated with these previously mentioned variables.

## Results

### Patients

The EPOS included 2294 patients. Their mean age was 62 (SD 12) years. The mean Karnofsky Performance score of this patient population was 59 (SD 17), meaning that most patients required some help, but could take care of most personal requirements [34]. The mean total Mini Mental State (MMS)-score was 27 (SD 3). On average it was 32 months (SD 46) since the cancer was diagnosed and five months (SD 11) since opioid treatment started. Men (52%) and women (48%) were equally represented in the study. The majority was hospitalized (81%), Caucasian (97%) patients with one or more metastases (83%). Cancer diagnoses, site of metastases and countries are given in Table 1.

**Table 1:** Demographics of patients

		Respondents (N = 1933)		Non respondents (N = 356)		p-value^a^
			
		N (%)	Mean (SD)	N (%)	Mean (SD)	
**Age**			61.6 (12.1)		65.7 (12.9)	< 0.001

**Karnofsky Performance Status (KPS)**			61.6 (16.4)		46.0 (16.0)	< 0.001

**Mini Mental State (MMS), total score**			26.9 (3.3)		25.7 (4.0)	0.003

**Time since diagnosis **(months)			32.8 (46.7)		25.3 (44.3)	0.001

**Time on opioids **(months)			5.2 (11.3)		3.1 (7.1)	0.002

**Gender**	Female	922 (47.7)		165 (47.1)		0.862
	Male	1011 (52.3)		185 (52.9)		

**Department**	Hospitalized patients	1513 (78.3)		338 (97.1)		< 0.001
	Out-patients	420 (21.7)		10 (2.9)		

**Status of opioid treatment**	Recently initiated, titration	353 (18.4)		94 (27.5)		< 0.001
	Stable dosing	1564 (81.6)		248 (72.5)		

**Metastases^b^**	Bone	875 (45.3)		146 (42.0)		0.266
	CNS	104 (5.4)		28 (8.0)		0.060
	Liver	466 (24.1)		95 (27.3)		0.223
	Lung	426 (22.0)		77 (22.1)		1.000
	Other	762 (39.4)		151 (43.4)		0.172

**Cancer diagnosis^b^**	Breast	268 (13.9)		33 (9.5)		0.025
	Female reproductive organs	145 (7.5)		29 (8.3)		0.584
	Gastrointestinal	442 (22.9)		83 (23.8)		0.730
	Haematological	113 (5.8)		20 (5.7)		1.000
	Head and neck	105 (5.4)		20 (5.7)		0.799
	Lung	346 (17.9)		71 (20.6)		0.257
	Other	168 (8.7)		40 (11.5)		0.106
	Prostate	230 (11.9)		34 (9.7)		0.275
	Unknown origin	51 (2.6)		11 (3.2)		0.591
	Urological	139 (7.2)		27 (7.7)		0.737

**Country**	Denmark	29 (1.5)		2 (0.6)		0.213
	Finland	30 (1.6)		0		0.010
	Germany	273 (14.1)		178 (50.0)		< 0.001
	Greece	5 (0.3)		0		1.000
	Iceland	145 (7.5)		5 (1.4)		< 0.001
	Italy	398 (20.6)		62 (17.4)		0.195
	Lithuania	54 (2.8)		0		< 0.001
	Norway	475 (24.6)		88 (24.7)		0.947
	Sweden	119 (6.2)		16 (4.5)		0.270
	Switzerland	114 (5.9)		1 (0.3)		< 0.001
	United Kingdom	291 (15.1)		4 (1.1)		< 0.001

**Reason for not completing all assessment forms**	Did not want to complete			96 (27.8)		
	Too ill			177 (51.3)		
	Unknown reason			72 (20.9)		

^a^P-values from Mann-Whitney U test or Fisher's Exact test (2 × 2-tables)

^b^Some patients had more than one cancer diagnosis and more than one localization of metastases, consequently the sum of percentages is larger than 100.

Three hundred and fifty-six patients answered less than 15 questions in the EORTC QLQ-C30 and were categorized as non-respondents. In addition observer rating of symptoms was missing for five patients, meaning that this study yielded 1933 patient and proxy assessment dyads. In general, patients not giving a self-report of symptoms were older, had lower Karnofsky Performance Status, lower scores on MMS, were more recently diagnosed with cancer, opioid treatment was more recently initiated and they were more often hospitalized as compared to those who completed the assessment form (Table 1). The most common reason for not completing all assessments was that the patients were too ill.

### Agreement between patients and health care providers

Nurses assessed the symptoms of 994 patients (51%), physicians assessed the symptoms of 735 patients (38%) and data about the health care provider profession was missing for 204 patients (11%). Health care providers systematically reported the percentages of moderate or severe symptoms as lower than the patients did. The percentages of patients with symptoms assessed as moderate or severe by patients and providers respectively, were for pain 67 and 47, for fatigue 71 and 54, for generalized weakness 65 and 47, for anorexia 47 and 25, for depression 31 and 17, for constipation 45 and 30, for poor sleep 32 and 21, for dyspnea 30 and 16, for nausea 27 and 14, for vomiting 14 and 6 and for diarrhea 14 and 6 (Table 2). Health care providers underestimated symptom intensity at the group level (p < 0.001 for all symptoms) (Table 2).

**Table 2:** Prevalence and intensity of symptoms as rated by patients and health care providers

		Symptom assessments					Wilcoxon signed-rank test^a^
			
Intensity		None	Mild	Moderate	Severe	Total	
**Absolute number (%)**		**N (%)**	**N (%)**	**N (%)**	**N (%)**	**N**	

**Pain**	Patient	124 (6.4)	508 (26.4)	799 (41.5)	492 (25.6)	1923	-18.5 ^b^
	Provider	253 (13.1)	761 (39.5)	691 (35.8)	223 (11.6)	1928	

**Fatigue^c^**	Patient	142 (7.4)	423 (21.9)	634 (32.8)	731 (37.9)	1930	-18.3 ^b^
	Provider	230 (11.9)	661 (34.3)	741 (38.5)	294 (15.3)	1926	

**Generalized weakness**	Patient	153 (8.0)	517 (27.0)	712 (37.1)	536 (27.9)	1918	-16.1 ^b^
	Provider	281 (14.6)	740 (38.3)	635 (32.9)	275 (14.2)	1931	

**Anorexia**	Patient	510 (26.5)	504 (26.2)	477 (24.8)	432 (22.5)	1923	-20.2 ^b^
	Provider	910 (47.4)	535 (27.9)	322 (16.8)	154 (8.0)	1921	

**Depression**	Patient	639 (33.4)	683 (35.7)	397 (20.8)	192 (10.0)	1911	-16.8 ^b^
	Provider	966 (50.1)	644 (33.4)	263 (13.6)	57 (3.0)	1930	

**Constipation**	Patient	583 (30.3)	476 (24.8)	471 (24.5)	392 (20.4)	1911	-17.2 ^b^
	Provider	856 (44.4)	487 (25.3)	393 (20.4)	192 (10.0)	1928	

**Poor sleep**	Patient	735 (38.2)	572 (29.7)	409 (21.2)	209 (10.9)	1925	-13.8 ^b^
	Provider	976 (50.8)	554 (28.8)	309 (16.1)	84 (4.4)	1923	

**Dyspnea**	Patient	801 (41.7)	552 (28.7)	362 (18.8)	208 (10.8)	1923	-18.5 ^b^
	Provider	1199 (62.1)	414 (21.5)	219 (11.3)	98 (5.1)	1930	

**Nausea**	Patient	871 (45.2)	530 (27.5)	342 (17.7)	184 (9.5)	1927	-18.0 ^b^
	Provider	1222 (63.3)	439 (22.7)	207 (10.7)	62 (3.2)	1930	

**Vomiting**	Patient	1317 (68.3)	351 (18.2)	161 (8.4)	99 (5.1)	1928	-16.2 ^b^
	Provider	1635 (84.7)	182 (9.4)	83 (4.3)	30 (1.6)	1930	

**Diarrhea**	Patient	1334 (69.6)	313 (16.3)	183 (9.5)	88 (4.6)	1918	-16.5 ^b^
	Provider	1664 (86.2)	157 (8.1)	84 (4.4)	26 (1.3)	1931	

^a^Wilcoxon signed-rank test calculations are based on rankings of the differences found by subtracting the patient's symptom score from the provider's symptom score. For all symptoms, the sum of the negative difference ranks was larger than the positive difference ranks indicating that health care providers tend to underestimate symptom intensity compared to patients.

^b^p < 0.001

^c ^Fatigue was originally assessed in EORTC QLQ-C30 with a scale ranging from 0 to 100. To ensure comparability with the four-point fatigue item assessed by providers, the fatigue scale was recoded into a four-point scale with increasing symptom intensity from 1 to 4: 0-24 was recoded into 1, 25-49 was recoded into 2, 50-74 was recoded into 3 and 75-100 was recoded into 4.

Direct under- and overestimations were calculated on the basis of difference scores. Again, underestimation of symptoms by health care providers was far more common than overestimation (Table 3). For instance anorexia was underestimated in 20 percent of assessment-pairs and overestimated in two percent. In a majority of patient-provider assessment pairs (79 to 93 percent) the responses were identical or differed by only one response category. The highest levels of agreement were found for pain, vomiting and diarrhea, where 90 percent of assessment-pairs showed complete agreement or differed by only one response category. Fatigue, anorexia and constipation were the symptoms most frequently underestimated by health care providers.

**Table 3:** Under- and overestimation of symptoms by health care providers as compared to patients

Symptom (difference)	Health care provider underestimation			Good agreement			Health care provider overestimation		
	
	-3	-2	%	0	± 1	%	2	3	%
**Pain**	28	148	9.2	904	822	90.0	16	0	0.8

**Fatigue**	32	226	13.4	795	838	84.9	30	2	1.7

**Generalized weakness**	26	178	10.6	781	883	86.8	44	4	2.5

**Anorexia**	127	243	19.4	865	636	78.5	33	8	2.1

**Depression**	50	149	10.4	1028	652	88.1	28	1	1.5

**Constipation**	66	156	11.6	1101	570	87.2	21	3	1.3

**Poor sleep**	46	150	10.2	1037	649	88.0	26	7	1.7

**Dyspnea**	41	153	10.1	1127	577	88.8	17	5	1.1

**Nausea**	50	149	10.3	1177	534	88.9	13	1	0.7

**Vomiting**	39	87	6.5	1445	346	93.0	8	0	0.4

**Diarrhea**	33	106	7.3	1465	307	92.5	4	1	0.3

Number and percentage of patient-provider pairs with symptom scores showing good agreement (health care provider minus patient score within ± 1), considerable underestimation (health care provider minus patient score ≤ -2) and considerable overestimation (health care provider minus patient score ≥ 2).

**Figure 1:**
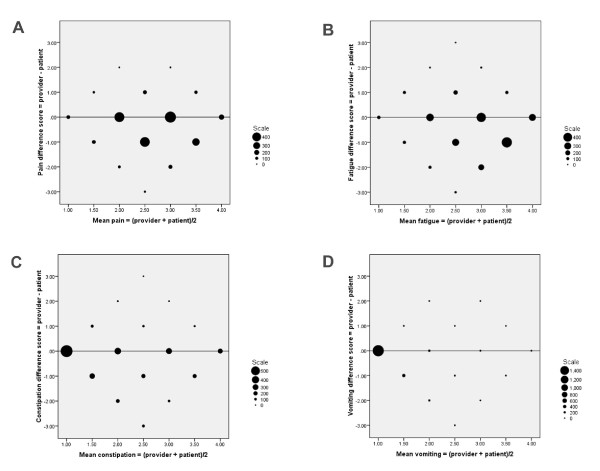
**Bland-Altman plots, one for each symptom (see also Figure 2)**. The difference between patient and provider score (difference score = health care provider score minus patient score) plotted against mean symptom score. The size of markers reflect the number of individual observations and only the line of equality (difference = 0) is shown. Negative differences mean that providers underestimated the symptom. The larger the size of the markers at one side of the line of equality, the larger was the tendency of a systematic difference between assessments (i.e. more observations below the line suggest that providers had a negative bias and underestimated symptom intensity). Whether differences between provider and patient assessments changes with the mean value of symptom intensity is determined by looking for patterns along the x-axis. **(A): **Pain. **(B): **Fatigue. **(C): **Constipation. **(D): **Vomiting.

**Figure 2:**
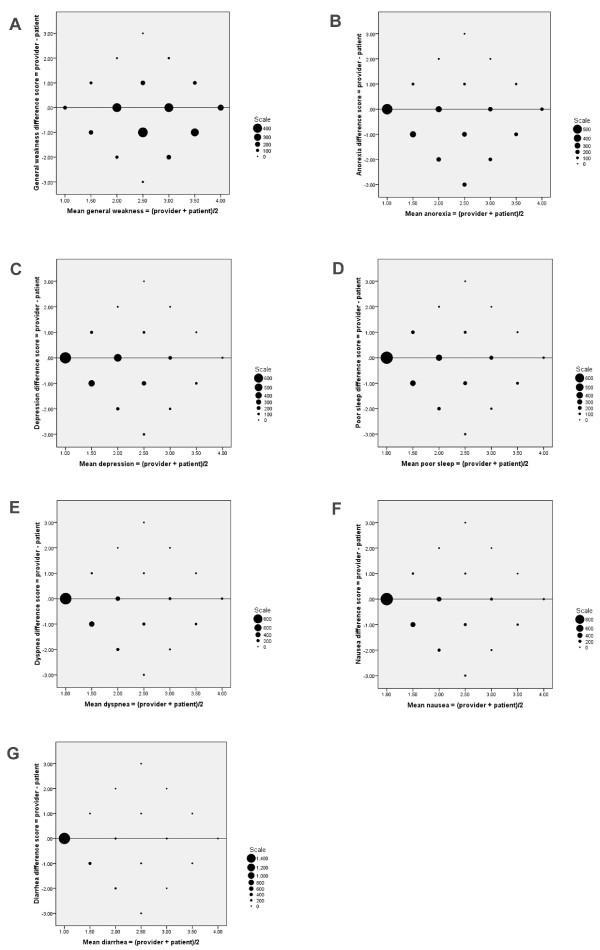
**Bland-Altman plots, one for each symptom (see also Figure 1)**. The difference between patient and provider score (difference score = health care provider score minus patient score) plotted against mean symptom score. The size of markers reflect the number of individual observations and only the line of equality (difference = 0) is shown. Negative differences mean that providers underestimated the symptom. The larger the size of the markers at one side of the line of equality, the larger was the tendency of a systematic difference between assessments (i.e. more observations below the line suggest that providers had a negative bias and underestimated symptom intensity). Whether differences between provider and patient assessments changes with the mean value of symptom intensity is determined by looking for patterns along the x-axis. **(A): **Generalized weakness. **(B): **Anorexia. **(C): **Depression. **(D): **Poor sleep. **(E): **Dyspnea. **(F): **Nausea. **(G): **Diarrhea.

At the individual level, the patient-provider agreement was evaluated for each symptom by the ICC (Table 4). Agreement for anorexia was poor to fair (ICC < 0.4), whereas the ICC of all other symptoms was of a moderate magnitude (ICC 0.4-0.6). The individual differences between the two assessments (difference score = health care provider score minus patient score) were assigned as the ordinate (y-axis) and the individual means as the abscissa (x-axis), in Bland-Altman plots (Figure 1 and Figure 2). The size of the markers reflect the number of individual observations and only the line of equality (difference = 0) is shown. The Bland-Altman plot of pain showed that the best agreement was found at intermediate levels of symptom intensity (Figure 1a). The Bland-Altman plots of fatigue and generalized weakness demonstrated increasing agreement with increasing symptom intensity (Figure 1b and 2a). For constipation, anorexia, depression and poor sleep (Figure 1c, 2b, c and 2d) the Bland-Altman plots showed a fair agreement at all levels of symptom intensities, with the majority of agreement-pairs within ± 1 difference scores. For the symptoms that were less frequent, like vomiting, dyspnea, nausea and diarrhea (Figure 1d, 2e, f and 2g) most patients and health care providers agreed on absence of the symptom.

**Table 4:** Difference scores and intraclass correlation coefficients for patient-provider-pairs of symptom severity scores

	Total	Difference score^a^		Intraclass correlation	
	
Symptom	N	Mean	SD	ICC^b^	95% CI
**Pain**	1918	-0.40	0.86	0.46	0.31 - 0.57

**Fatigue**	1923	-0.44	0.97	0.40	0.27 - 0.51

**Generalized weakness**	1916	-0.38	0.96	0.41	0.31 - 0.50

**Anorexia**	1912	-0.58	1.10	0.38	0.22 - 0.51

**Depression**	1908	-0.38	0.90	0.46	0.33 - 0.55

**Constipation**	1917	-0.39	0.92	0.59	0.47 - 0.68

**Poor sleep**	1915	-0.31	0.93	0.49	0.41 - 0.56

**Dyspnea**	1920	-0.39	0.85	0.56	0.41 - 0.66

**Nausea**	1924	-0.38	0.84	0.53	0.39 - 0.63

**Vomiting**	1925	-0.28	0.70	0.51	0.40 - 0.60

**Diarrhea**	1916	-0.28	0.69	0.51	0.39 - 0.60

^a^Difference scores were calculated by subtracting the patient's symptom score from the health care provider's symptom score.

^b^Intraclass correlation coefficient, two way mixed effect model; absolute agreement definition; single measure ICC.

### Factors associated with symptom agreement

The 1933 patient-provider dyads assessing 11 symptoms yielded 21 263 paired symptom observations. The total number of dyads differed somewhat due to missing data (Table 5). For all symptom assessment pairs, the agreement between patients and their health care provider was not associated with the patients' age, gender, BMI, previous abuse of alcohol, use of chemotherapy during the past 24 hours or presence of metastatic disease (Table 5). Karnofsky Performance Status had a substantial effect on agreement. Large patient-provider discrepancies were more prevalent in patients with a very low (≤ 25) or a moderately impaired (51 to 75) performance status. Higher MMS-scores (> 23) were associated with more underestimation of symptom intensity by health care providers as compared to patients with lower MMS-scores (≤ 23). Health care providers more often underestimated symptoms in recently diagnosed patients and patients that received a recently initiated opioid still being titrated. Patient-provider agreement was better among outpatients than hospitalized patients. Overestimation of symptoms by health care providers was more common in patients with a history of drug abuse (3%) than in patients with no such history (1%). Both under- and overestimation was more common when the symptoms were assessed by physicians (11% and 3% respectively), as compared to assessment by nurses (10% and 2% respectively). The frequency of underestimation varied between cancer diagnoses and countries (Table 5).

**Table 5:** Factors associated with agreement across all pairs of symptom observations (N = 21263)^a^

Factors possibly affecting agreement		Health care provider underestimation		Good agreement		Health care provider overestimation		Chi-square
		
		Number	%	Number	%	Number	%	
**Age**	≤ 60	1013	10.9	8122	87.7	126	1.4	0.629
	> 60	1268	10.7	10410	88.0	146	1.2	

**Body Mass Index**	< 25	1378	10.3	11855	88.5	169	1.3	0.583
	≥ 25	693	9.8	6274	88.9	89	1.3	

**KPS^b^**	< 80	1808	11.1	14110	86.8	347	2.1	0.032
	≥ 80	474	9.9	4240	88.2	94	2.0	

**Mini Mental State,** **total score**	≤ 23	265	9.5	2455	87.6	81	2.9	< 0.001
	> 23	1927	11.3	14806	86.7	340	2.0	

**Time since** **diagnosis **(months)	≤ 3	550	12.5	3797	86.5	43	1.0	< 0.001
	> 3	1420	9.6	13162	89.0	204	1.4	

**Gender**	Female	1122	11.1	8812	87.5	136	1.4	0.261
	Male	1161	10.5	9727	88.2	136	1.2	

**Department**	Hospitalized patients	1992	12.1	14314	86.7	201	1.2	< 0.001
	Out-patients	291	6.3	4225	92.1	71	1.5	

**Status of** **opioid treatment**	Recently initiated	593	15.4	3219	83.5	43	1.1	< 0.001
	Stable dosing	1679	9.8	15159	88.6	228	1.3	

**Previous or ongoing** **abuse alcohol**	Yes	113	9.8	1017	88.5	19	1.7	0.304
	No	2161	10.9	17477	87.9	252	1.3	

**Previous or ongoing** **abuse drugs**	Yes	19	9.2	180	87.4	7	3.4	0.024
	No	2250	10.8	18283	87.9	267	1.3	

**Chemotherapy the** **past 24 hours**	Yes	331	10.4	2833	88.6	32	1.0	0.180
	No	1952	10.9	15706	87.8	240	1.3	

**Profession of health** **care provider^c^**	Physician	859	10.6	6970	86.2	261	3.2	< 0.001
	Nurse	1090	10.0	9573	88.1	208	1.9	

**Metastases**	None	406	11.2	3176	87.7	39	1.1	0.430
	One or more	1917	11.0	15352	87.7	233	1.3	

**Cancer diagnosis**	Breast	292	10.0	2609	89.0	31	1.1	< 0.001
	Female reproductive organs	183	11.5	1383	87.2	20	1.3	
	Gastrointestinal	455	9.4	4306	89.4	58	1.2	
	Haematological	142	11.5	1077	87.3	14	1.1	
	Head and neck	148	12.8	983	85.3	21	1.8	
	Lung	481	12.8	3241	86.0	46	1.2	
	Other	198	10.8	1607	87.6	29	1.6	
	Prostate	237	9.4	2233	89.0	40	1.6	
	Unknown origin	53	9.6	491	89.3	6	1.1	
	Urological	171	11.4	1317	87.5	18	1.2	

**Country**	Denmark	3	0.9	304	95.3	11	3.5	< 0.001
	Finland	25	7.7	295	90.8	5	1.5	
	Germany	662	22.4	2231	75.5	61	2.1	
	Greece	5	9.3	48	88.9	1	1.9	
	Iceland	105	6.6	1437	90.3	49	3.1	
	Italy	137	3.1	4196	96.1	34	0.8	
	Lithuania	44	7.5	533	90.5	12	2.0	
	Norway	546	10.5	4578	88.4	54	1.0	
	Sweden	182	14.0	1107	85.3	9	0.7	
	Switzerland	281	22.8	937	76.1	13	1.1	
	United Kingdom	293	9.2	2874	89.8	33	1.0	

^a^Number of paired symptom observations. Across one pair of raters and 11 questions for 1933 patients (1 × 11 × 1933 = 21263) (varies due to missing data).

^b^Karnofsky performance status

^c^For 204 patients, the profession of the health care provider was not reported.

## Discussion

In this large, European cross-sectional study a moderate agreement on symptom assessment between cancer patients and their health care providers was observed (ICC range 0.38 to 0.59). Health care providers underestimated symptom intensity in approximately 10% of patients, with some variations between cancer diagnoses and substantial variations between countries. Patients with low Karnofsky Performance Status, MMS-scores of 24 or higher, who were hospitalized, recently diagnosed or still undergoing opioid titration, were at increased risk of symptom underestimation by health care providers.

Our examinations of the agreement between patient and provider assessments showed that health care providers tended to underestimate all symptoms and that underestimation was present in 6.5 to 19.4% of the patient-provider assessment pairs. The highest rates of underestimation were found for anorexia (19.4%) and fatigue (13.4%), which were among the most "subjective" symptoms investigated. As similar proportions of underestimation have been demonstrated previously [15,18,28,29], influence from subjective terminology and recoding of the fatigue scale was considered as less likely and the findings may rather reflect the known trend towards better agreement when the information assessed is observable and concrete [14]. This was also illustrated by the low level of underestimation (6.5%) for vomiting. Agreement at the individual level was only moderate as measured by ICCs ranging between 0.38 and 0.59. The ICC, which is a suitable statistical measure of agreement correcting for the chance-expected agreement, and the presentation of agreement as absolute numbers and percentages in Table 3 are complementary approaches as they differ both in perspective and operationalization [27].

The present findings of provider underestimation and moderate ICCs were in contrast to findings in primary care where general practitioners and nurses overestimated symptoms and ICCs were higher [19]. Also, in a review of health care providers' role for evaluation of patients with chronic diseases including cancer, Sneeuw et al. reported that providers tended to rate patients as having more symptomatology than the patients did themselves [15]. However, the quality of the studies comparing patient and provider assessments was assessed as rather poor [15]. Other studies have reported that health care providers tend to underestimate physical symptoms [4,20,23,24,28], whereas anxiety, depression and distress of symptoms are overestimated [14]. In the present study there was no directional difference between agreement on physical and psychological symptoms. However, the Bland-Altman plots of agreement by level of symptom intensity showed differences between symptoms. For fatigue and generalized weakness, the agreement increased at higher symptom intensities, whereas the agreement on less prevalent symptoms was best at low symptom intensities. The latter finding was in line with observations in primary care where symptom agreement was best for absent symptoms [19]. This argues for that the differences in agreements between studies are likely to be influenced by the number of patients with moderate or severe symptoms included in the study.

Symptom underestimation by health care providers was associated with low Karnofsky Performance Status, high MMS-score, hospitalization, recently diagnosed cancer and ongoing opioid titration. Of the previous studies examining possible factors associated with patient-provider agreement one study found no associations [18], and three studies found better agreement in subgroups of patients characterized by certain demographic- and disease-related factors [28-30]. However, the findings were not conclusive because the analyses were dispersive, the findings were difficult to explain clinically and there was a lack of consistency. The significant relationship between Karnofsky Performance Status and agreement found in the present study was in line with the existing literature, but there was no linear [28,29] or U-shaped [11,27] correlations as described previously. To our knowledge, an association with MMS-score has not been documented before. The association might reflect that patients with low MMS-scores are identified as generally more influenced by disease and therefore the observer ratings agreed more closely with the patient ratings. The status of patients hospitalized, recently diagnosed or undergoing opioid titration can at a group level be considered as fluctuating. Therefore, the finding of more discrepancies between patient and provider assessments in these patients was not surprising. This finding is also in line with a previous study showing less agreement in hospitalized patients [30]. Based upon these results the presence of such factors could alert clinicians to recognize that health care providers may underestimate symptoms in these patients. The factors associated with agreement were broadly similar to those characterizing the patients not able to complete the EORTC questionnaire, possibly indicating that patients unable to give self-reports are at risk of underestimation of symptoms by health care providers.

Drug abuse and cancer diagnosis were also significantly related to agreement, but the magnitude of differences was considered as not clinically important. As seen in previous studies, the nurse assessments agreed more closely with patient ratings than physician assessments [4,19]. The substantial variations in agreement observed between countries might be of relevance, reflecting that factors influencing either patient- or provider reports may vary between countries. However, the study design does not allow us to reach a firm conclusion if this variation is truly related to country or if it also related to each specific center.

For the analyses of agreement, the patient ratings were used as a "gold standard to which the health care provider assessments were compared. In reviews, the patients' role as a gold standard for assessment has been questioned [15,40]. A study comparing responses from patients, physicians, nurses and significant others found that deviant scores of more than one response category were most often caused by the patients, indicating either that the quality of all proxy-derived information was poor or that patients' responses are of questionable validity [27]. Patient reports may not reflect the true experience of symptoms for various reasons, such as psychological denial, barriers towards reporting symptoms, a wish to please the nurse/doctor or an impression that emphasizing symptoms is needed to secure help from health care providers. Therefore, to use a combination of patient reports (when available), the caregiver's perceptions and objective signs could be of benefit both in clinical decisions and future research [4,7].

The findings of this study might have direct clinical implications for the care of cancer patients. Underestimation of symptoms by health care providers in cancer care might cause undertreatment of symptoms and less favorable outcome. Insight into the limitations of observer rating may improve the health care providers' ability to identify symptoms that need treatment. To increase rates of agreement, systematic screening tools used regularly or programs for further training of communication skills could be introduced [41]. Future research could address the consequences of symptom underestimation in cancer care and the importance of interventions to reduce disagreement between patients and providers. When health care provider assessments replace patient rating in those not able to report their symptoms, the identification of risk factors for erroneous assessments by health care providers is important [24]. Our findings of factors influencing agreement may be elaborated in future studies investigating whether agreement can be improved by adjusting for systematic deviations between patient and health care provider assessments in such situations.

This is to our knowledge the largest study comparing the assessment of individual symptoms between patients and health care providers in cancer care across several countries. As in other studies among advanced cancer patients, self-assessment was not possible to obtain for a considerable number of patients (N = 365). The patients not able to report symptoms had similar characteristics as those who had their symptoms underestimated by providers. Therefore, the true number of patients being underestimated is not likely to be falsely overestimated due to missing values. Patients rated their symptoms in the extensively validated EORTC QLQ-C30, whereas health care providers rated the same symptoms using a clinically applicable four-point verbal rating scale. Patients reported their symptoms during the past week, whereas health care providers assessed symptoms during the past 24 hours, a difference in time frame that might introduce differences in both prevalence and intensity of reported symptoms. Thus, it could be argued that patient- and provider-instruments differed. However, to compare the actual assessments currently performed in clinical research it was decided to apply the instrument used in most studies on self-reported symptoms in cancer care (EORTC QLQ-C30) and the bedside observer assessment tool used in recent European Association for Palliative Care (EAPC) endorsed studies [42]. The present study compared assessments by patients and providers and was not designed to compare assessments performed by subgroups of health care providers (nurses versus physicians). Future studies needs to be designed specifically to investigate differences between nurses and physicians in terms of ability to assess patient symptoms as the findings of previous studies are inconsistent [4,19,29]. Furthermore, studies in samples of other racial/ethnic composition and studies including patients at different levels of the disease-trajectory are needed to determine the generalizability of the findings of the present study.

We recognize that this study have some limitations. In the analyses to identify risk factors of disagreement between patients and providers, pairs of assessments for all symptoms were pooled, to avoid multiple testing. This strategy did not allow us to address whether the factors associated differently with each symptom. Furthermore, data from each center on numbers and characteristics of patients not approached or declining to take part in the study was not obtained. However, the characteristics of patients included were found to be representative for cancer patients. The pooling of data across countries may be seen as a limitation as there were substantial variations in agreement between countries, but it may also be a strength as it increase the sample size and protects against the tendency towards report of lower overall levels of patient-proxy agreement shown in smaller studies [15]. The selection of centers in each country was based upon researchers volunteering to participate in this European multi-center study. These centers are not necessarily representative for each country's general health care system. In order to accurately describe the influence of country, we believe a study should include several randomly selected centers from each country in the analysis. Obviously, this was not done in our study and therefore, we believe that the data about country effect (Table 5) should be interpreted with caution and hence not included in more comprehensive analyses.

## Conclusions

In this large European study, health care providers assessing cancer patients' symptoms tended to underestimate symptom intensity at the group level and the agreement with patient ratings at the individual level was moderate. The differences between patient and provider assessments can be caused by providers not being able to exactly interpret the patients' symptoms or that different instruments are used for patients and health care providers. Agreement on rating of symptoms was associated with demographic- and disease-related factors. Clinicians involved in care for patients with cancer should be aware of the potential factors associated with a risk of symptom underestimation.

## Competing interests

The authors declare that they have no competing interests.

## Authors' contributions

All authors contributed to and have approved the final manuscript. EAL conceived of the study and participated in its design, analyzed and interpreted data, and thereafter drafted the manuscript. MAGS was involved in drafting the manuscript and revising it critically for important intellectual content. KB was involved in acquisition and collection of data and revised the manuscript critically. FS participated in the design of the study and revised the manuscript critically. SK conceived of the study, participated in its design and revised the manuscript critically. PK was involved in conceiving, designing and coordinating the study, drafting the manuscript and revising it critically.
